# Treatment of Cerebral Vasospasm after Aneurysmal Subarachnoid Hemorrhage Using the Compliant Manually Adjustable Mesh Comaneci

**DOI:** 10.5334/jbsr.3714

**Published:** 2024-10-17

**Authors:** Adrien Guenego, Hamza Adel Salim, Maud Wang, Jeremy J. Heit, Niloufar Sadeghi, Noémie Ligot, Valentina Lolli, Fadi Tannouri, Fabio Silvio Taccone, Boris Lubicz

**Affiliations:** 1 Interventional Neuroradiology Department Hôpital Universitaire de Bruxelles (HUB), Brussels, Belgium; 2 Department of Radiology, Division of Neuroradiology, Johns Hopkins Medical Center, Baltimore, Maryland, USA; 3 Interventional Neuroradiology Department Hôpital Universitaire de Bruxelles (HUB), Brussels, Belgium; 4 Departments of Radiology and Neurosurgery, Stanford Medical Center, Palo Alto, California, United States of America; 5 Department of Radiology and Neuroradiology, Hôpital Universitaire de Bruxelles (HUB), Brussels, Belgium; 6 Department of Neurology, Hôpital Universitaire de Bruxelles (HUB), Brussels, Belgium; 7 Department of Radiology and Neuroradiology, Hôpital Universitaire de Bruxelles (HUB), Brussels, Belgium; 8 Department of Interventional Radiology, Hôpital Universitaire de Bruxelles (HUB), Brussels, Belgium; 9 Department of Intensive Care, Hôpital Universitaire de Bruxelles (HUB), Route de Lennik, 808, 1070 Brussels, Belgium; 10 Interventional Neuroradiology Department Hôpital Universitaire de Bruxelles (HUB), Brussels, Belgium

**Keywords:** vasospasm, ruptured aneurysm, subarachnoid hemorrhage, angioplasty, medical device

## Abstract

*Background and purpose:* Cerebral vasospasm (CV) following aneurysmal subarachnoid hemorrhage (aSAH) may lead to morbidity and mortality. Endovascular mechanical angioplasty may be performed if symptomatic CV is refractory to noninvasive medical management. Compliant and noncompliant balloons and manually adjustable mesh may be used in this indication. We describe our initial experience with the Comaneci (Rapid Medical, Yokneam, Israel) in cerebral vasospasm treatment following aSAH.

*Methods:* All patients included in the prospective observational SAVEBRAIN PWI (NCT05276934 on clinicaltrial.gov) study who underwent cerebral angioplasty using the Comaneci device for the treatment of medically refractory and symptomatic CV after aSAH were identified. Patient demographic information, procedural details, and outcomes were obtained from electronic medical records.

*Results:* Between February 2022 and June 2023, seven consecutive patients underwent CV treatment with the Comaneci. Angioplasty of 37 arterial segments (supraclinoid internal carotid artery, A1, A2, and A3 segments of the anterior cerebral artery and M1 and M2 segments of the middle cerebral artery) was attempted, and 35/37 (95%) were performed. The vessel diameter improved significantly following angioplasty (+64%), while brain hypoperfusion decreased (−45% of the mean *T*_Max_). There was no long-term clinical complication, and 6% per-procedural complications occurred.

*Conclusions:* The Comaneci is effective in the treatment of cerebral vasospasm following aSAH, bringing a new device in the armamentarium of the neurointerventionalist to perform intracranial angioplasty.

## Introduction

Aneurysmal subarachnoid hemorrhage (aSAH) remains a critical medical condition with significant morbidity and mortality, affecting approximately 40% of patients during the acute phase or within the first few weeks following aneurysm rupture [1]. The primary cause of morbidity in these patients is delayed cerebral ischemia (DCI) and cerebral vasospasm (CV) post‑aneurysm treatment. Despite effective initial management [2], many patients under the age of 65 remain dependent in their daily life activities [3, 4].

DCI is a prevalent neurological syndrome that impacts one‑third of patients with aSAH [5] and can lead to acute ischemic stroke if not managed effectively [6]. The pathophysiology of DCI is complex, but CV is a key contributor, occurring in 70% of aSAH cases. Of those who develop CV, 50% progress to DCI, resulting in either stroke or death [7].

Preventing and treating DCI primarily involves oral nimodipine and medical management, including vasopressor administration to increase arterial pressure if needed [8]. However, there is considerable variability [9] among neurointerventionalists regarding the endovascular management of vasospasm [10, 11]. There is no standardized approach to endovascular interventions for vasospasm, and the use of mechanical angioplasty is debated between stent retrievers and balloons. The effectiveness of mechanical angioplasty is also perceived differently within the community.

In this context, our study aims to report our initial experiences using a compliant manually adjustable mesh, the Comaneci device (Rapid Medical, Yokneam, Israel) for the treatment of medically refractory and symptomatic CV following aSAH.

## Methods

All patients were included in a prospective single‑center observational study, the SAVEBRAIN PWI study (NCT05276934 on clinicaltrial.gov). The study protocol was approved by the institutional review board and complied with the Health Insurance Portability and Accountability Act (HIPAA). Patient informed consents were signed prospectively for the SAVEBRAIN PWI study, and an analysis of these anonymized data was made.

### The SAVEBRAIN PWI study

The SAVEBRAIN PWI (NCT05276934 on clinicaltrial.gov, brain imaging after nontraumatic intracranial hemorrhage) is an ongoing prospective monocenter observational noninterventional study gathering data from patients with CV following aSAH. Follow‑up and treatments were performed according to our local protocols. Primary outcome parameters include changes in perfusion parameters on CTP before and after treatment and changes in post‑treatment vessel caliber when patients are treated endovascularly for CV refractory to standard hospital care.

Our local management protocol is as follows: (1) initial diagnosis of SAH based on computed tomography (CT), CT angiography (CTA), and CT perfusion (CTP), followed by DSA; (2) external cerebrospinal fluid and/or parenchymal hematoma drainage, if deemed necessary by the on‑call neurosurgical team; (3) endovascular aneurysmal treatment as a first‑line treatment (microsurgical aneurysm treatment in rare cases) based on an individualized multidisciplinary decision process; (4) per os administration of nimodipine on the day of admission; and (5) daily transcranial Doppler and clinical examination, as well as brain CTP at day 4 and 10, and in case of CV refractory to medical noninvasive treatment.

### Population

We identified all patients who underwent an endovascular treatment for CV by mechanical angioplasty from February 2022 to June 2023 and met the following criteria: (1) aSAH complicated by a symptomatic CV defined as an angiographic vascular spasm/stenosis; (2) CV refractory to medical noninvasive treatment in the intensive care unit, leading to multidisciplinary talks between the intensivist and the neurointerventionalist and decision to perform an endovascular treatment of those patients; and (3) mechanical angioplasty performed or attempted using a Comaneci in the anterior circulation. The decision to perform the endovascular procedure was made by consensus on an individual patient basis, between the neurointerventionalist, intensive‑care physician, and neurosurgeon; the final technical aspects were left at the discretion of the treating neurointerventionist physician. The procedures were carried out using standard practices and recommendations. The patient’s baseline clinical and radiological characteristics, procedure details, and outcomes were collected using standardized definitions [12]. All cases were reviewed by two board‑certified neuroradiologists (A.G. with 9 years of neuroradiology experience and M.W. with 4 years of neuroradiology experience); they determined the angiographic treatment success. In cases of inconsistency concerning the final result, a decision was made by consensus. All cases of mechanical angioplasty performed in the posterior circulation were not included in the study as there are very limited data supporting the value of CT in these arteries. Furthermore, sensitivity to brain stem ischemia and hypoperfusion are different than to supratentorial brain because of limited spatial resolution for small posterior fossa infarcts and artifacts [13, 14].

### Patient and public involvement

Patients, industry, or the public were not involved in the design, conduct, reporting, or dissemination plans of our research.

### Procedures

All procedures were performed in a dedicated neuroangiography suite with a Philips biplane system. The procedures were performed under general anesthesia. The common femoral artery was accessed with a short (10 cm) 5F or 6F vascular sheath (Cordis, Bridgewater, New Jersey, USA); baseline diagnostic cerebral angiograms were performed with a 5F or 6F ENVOY DAXB (Cerenovus, Irvine, California, USA). The catheter was positioned in the affected internal carotid artery (ICA), and IA nimodipine was infused according to our local protocols at a rate of 12 mg/H for a period of 10 min (2 mg of nimodipine per affected ICA) before and during the angioplasty. Angioplasty was performed with the Comaneci in patients with persisting moderate or severe vasospasm. A Comaneci 17 was used for the M1, M2, A1, A2, and A3 segments; the Comaneci was used for the M1, M2, A1, and A2 segments; and the Comaneci Petit was used for the M1, M2, and supraclinoid ICA segments. Intravenous heparin was not routinely administered.

The Comaneci was used according to the manufacturer’s instructions, navigating a 14‑inch microwire, a 17 microcatheter was navigated through the guiding catheter into the affected artery and the Comaneci was deployed. Under live fluoroscopy, the device was slowly opened until achieving a full opening. The Comaneci 17 was left at that diameter for approximately 60 s, after which it was closed. It was then navigated into more proximal affected branches.

The patient’s blood pressure was monitored by anesthesiologists using an arterial line to maintain mean arterial pressure > 90 mmHg. After IA nimodipine infusion, the Comaneci was advanced into the affected intracranial vessels, and the angioplasty was performed as described.

### Compliant manually adjustable mesh

The Comaneci device is a “compliant radiopaque mesh composed of 12 nitinol wires mounted on a core wire with three versions currently available. The standard Comaneci version has a diameter of 1.5 mm–4.5 mm with a length ranging from 32 mm to 12 mm when fully deployed. The Comaneci Petit has a diameter of 1.5 mm–3.5 mm and a length of 24 mm to 21 mm. Both need to be delivered through a 0.021‑inch microcatheter” [15]. The Comaneci 17 can be delivered through a 0.017‑inch microcatheter, with diameters ranging from 0.5 to 3.0 mm and a length of 22 mm to 16 mm [16]. Each increment increases the diameter of the device while also decreasing its length.

### Statistical analyses

The objectives were to analyze the trackability of the device, measured by the percentage of successfully catheterized intracranial vessels, then its efficacy [evolution in vessel diameter (vessel diameter was measured on the exact same incidence where the Comaneci was deployed, before and after deployment using two‑dimensional [2D] images from the DSA. Measurements were made using the maximum zoom to have the highest precision possible) and in CT perfusion parameters between before and after angioplasty, need of retreatment of the vessel] and safety (rate of periprocedural complications, overinflation of the balloon, and visibility of the inflated balloon during the procedure rated as adequate or inadequate by the treating neurointerventionalist). Continuous variables are reported as mean (± SD) or median [interquartile range (IQR)]. Categorical variables are reported as proportions. All statistical analyses were performed with XLSTAT (Addinsoft, New York City, NY).

## Results

Seven consecutive patients (five women and two men) (see Supplementary Files) were identified who underwent endovascular mechanical angioplasty with the Comaneci for CV. The median age of the patients was 54 (47–64) years. All patients presented with Fisher grade IV hemorrhage due to ruptured aneurysms arising from the anterior communicating artery (3/7, two of them were treated by coiling remodeling, one was clipped at day 1, but as there was no complete occlusion, we decided to treat the remnant using coiling remodeling), V4/posteroinferior cerebellar artery (2/7, one was considered as a distal PICA dissecting aneurysm, coiling remodeling was attempted but failed, and clipping was discussed, but the PICA and the ruptured aneurysm were finally occluded using glue; the second aneurysm was located at the PICA bifurcation and was treated by coiling remodeling), superior cerebellar artery (1/7, was treated by coiling remodeling), or posterior communicating artery (1/7, was treated by coiling remodeling). Aneurysms were right sided in 4/7 (57%). All patients suffered from an initial acute hydrocephalus and were treated with an external ventricular drain. All patients were treated by oral nimodipine as recommended (class I, level A) [17].

Five of these aneurysms were treated by coiling remodeling (71%), and one aneurysm was treated by clipping then coiling for a remnant (14%), while one distal dissecting aneurysm was treated by glue embolization (14%). The clinical characteristics are summarized in Table 1.

**Table 1:** Baseline characteristics.

VARIABLE	ALL
**Number of patients (%)**	7/7 (100%)
**Number of segments (%)**	35/35 (100%)
**Age, years (median, IQR)**	54 (47–64)
**Female (%)**	5/7 (71%)
**Medical history**	
**Untreated high blood pressure (%)**	1/7 (14%)
**Diabetes (%)**	0/7 (0%)
**Hyperlipidemia (%)**	1/7 (14%)
**Antiplatelets/anticoagulation (%)**	0/7 (0%)
**Current smoking (%)**	2/7 (28%)
**Prestroke mRS of 0–2 (%)**	7/7 (100%)
**WFNS Scale (median, IQR)**	5 (3–5)
**Hunt and Hess Scale (median, IQR)**	5 (3–5)
**Glasgow Coma Scale (median, IQR)**	3 (3–10)
**Aneurysm diameter, mm (median, IQR)**	3.9 (2.7–5.8)
**Aneurysm height, mm (median, IQR)**	6.0 (3.5–7.6)
**Aneurysm neck, mm (median, IQR)**	3.8 (2.6–5.1)
**Coiling remodeling (%)**	5/7 (71%)
**Imaging and presentation**	
**mFisher Scale (median, IQR)**	4 (4–4)
**Hydrocephalus (%)**	7/7 (100%)
**Parenchymal hematoma (%)**	4/7 (57%)
**External ventricular drain (%)**	7/7 (100%)
**Craniectomy (%)**	2/7 (28%)
**Before treatment *T*_Max_, sec (median, IQR)**	3.9 (3.6–4.1)
**Baseline artery diameter, mm (median, IQR)**	2.5 (1.9–2.8)
**Before treatment artery diameter, mm (median, IQR)**	1.3 (1.2–1.6)
**Before treatment visual vasospasm**	
**Moderate (%)**	18/35 (52%)
**Severe (%)**	17/35 (48%)

*Continuous variables are reported as mean (± SD) or median [interquartile range (IQR)].

**Categorical variables are reported as proportions.

All patients were intubated with sedation and ventilation, refractory CV was diagnosed by trans‑cranial Doppler, CT perfusion, and invasive parameters worsening. The patients had significant intracranial spastic stenosis on CTA and DSA. These data are summarized in Table 2.

**Table 2:** Procedural characteristics and outcomes.

VARIABLE	ALL
**Number of treated patients (%)**	7/7 (100%)
**Number of treated segments (%)**	35/35 (100%)
**Segment localization**	
**Carotid T (%)**	2/35 (6%)
**M1 (%)**	8/35 (23%)
**M2 (%)**	9/35 (25%)
**A1 (%)**	6/35 (17%)
**A2 (%)**	8/35 (23%)
**A3 (%)**	2/35 (6%)
**Left side (%)**	15/35 (43%)
**General anesthesia (%)**	33/35 (94%)
**Intra**v**enous heparin during treatment (%)**	4/35 (11%)
**Intra‑arterial chemical treatment, mg (median, IQR)**	2 (2–3)
**Intra‑arterial chemical treatment, min (median, IQR)**	10 (10–15)
**Imaging outcomes**	
**Post treatment *T*_Max_, seconds (median, IQR)**	1.4 (1.2–1.6)
**Post treatment artery diameter, mm (median, IQR)**	2.1 (2.0–2.6)
**Post treatment visual vasospasm (%)**	
**None (%)**	31/35 (89%)
**Moderate, no further treatment needed (%)**	4/35 (11%)
**Artery diameter improvement in percent [after/before, median IQR)]**	64 (48–113)
***T*_Max_ change in percent [after/before, median (IQR)]**	−45 (−40 to −52)
**Need to retreat the dilated segment (%)**	19/35 (54%)
**Days to retreatment, (median, IQR)**	2 (2‑3)
**Complications**	
**No complication (%)**	2/35 (6%)
**Post‑treatment CT extravasation (%)**	0/35 (0%)
**Post‑treatment cervical dissection (%)**	0/35 (0%)
**Post‑treatment distal clots (%)**	2/35 (6%)

*Continuous variables are reported as mean (± SD) or median [interquartile range (IQR)].

**Categorical variables are reported as proportions.

A median of 2 (2–3) mg of IA nimodipine were administered in each ICA prior and during the angioplasty in all patients. Multiple cerebral vessel segments were treated by angioplasty with the Comaneci, 35/37 (95%) segments were successfully catheterized (Table 2). The 2/37 (5%) segments we did not manage to catheterize were one distal middle cerebral artery M3 segment and one distal anterior cerebral artery A3 segment, using a 0.017” microcatheter.

All angioplasty procedures resulted in a markedly improved caliber of the treated vessels [+64% of diameter improvement (median of 2.1 [2.0–2.6] mm after treatment compared with 1.3 mm [1.2–1.6] before treatment) compared with before treatment] with either minimal or no residual vasospasm identified, with a corresponding CT perfusion improvement in all patients [*T*_Max_ decreased respectively by 45% (median of 1.4 [1.2–1.6] s after treatment compared with 3.9 s [3.6–4.1] before treatment)]. Afterwhile, 59% (19/35) of the treated segments needed a retreatment, and in case of retreatment, it happened after a median of 2 (2–3) days.

There was no long‑term clinical complication, 6% (2/35) per‑procedural complications occurred; one patient experienced distal A2–A3 small clots and an second patient experienced distal M4 clots while retrieving the Comaneci and microcatheter, and curative anticoagulation was started for both, blood pressure was increased, the main catheter was withdrawn, and a new catheter was used to make control runs. After 30 min, no significant residual clot or parenchymal defect were seen for both patients and the procedures were stopped. No patient died during or after the procedure.

**Figure 1:**
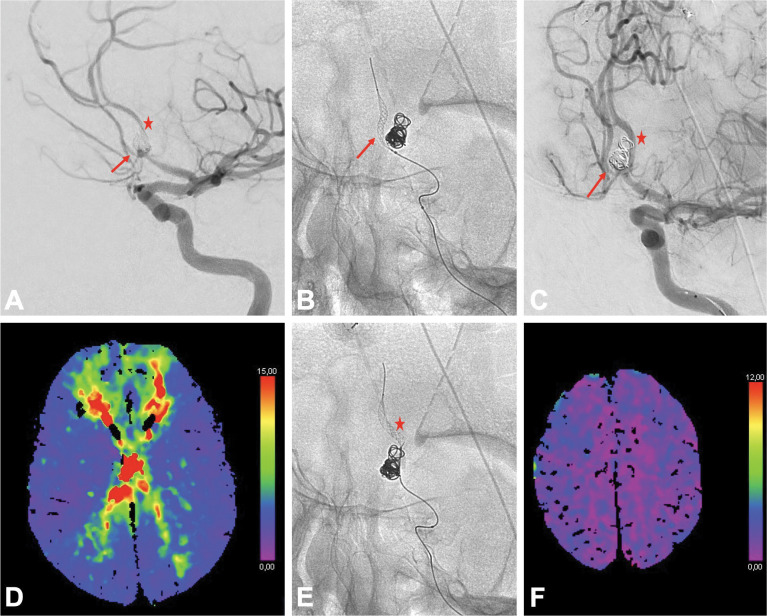
Cerebral angioplasty of the anterior cerebral artery (ACA) using the Comaneci device in a patient with an anterior communicating artery aneurysm treated by coiling remodeling previously, and an hypoplastic right A1 segment. **A.** Cerebral angiogram prior to endovascular treatment of the vasospasm demonstrating severe vasospasm of the left A1, left A2 (star), and right A2 segments (arrow). **B.** Fluoroscopic image demonstrating angioplasty of the right A2 segment with the Comaneci over a 0.0165” microcatheter (arrow). **C.** Final cerebral angiogram following angioplasty demonstrating an improved caliber of the left A1, left A2 (star), and right A2 segments (arrow). **D.** Axial *T*_Max_ map before treatment showing a severely delayed *T*_Max_ for both ACA territory. **E.** Fluoroscopic image demonstrating angioplasty of the left A2 segment with the Comaneci (star). **F.** Axial *T*_Max_ map following treatment showing an improved *T*_Max_ for both ACA territory.

**Figure 2:**
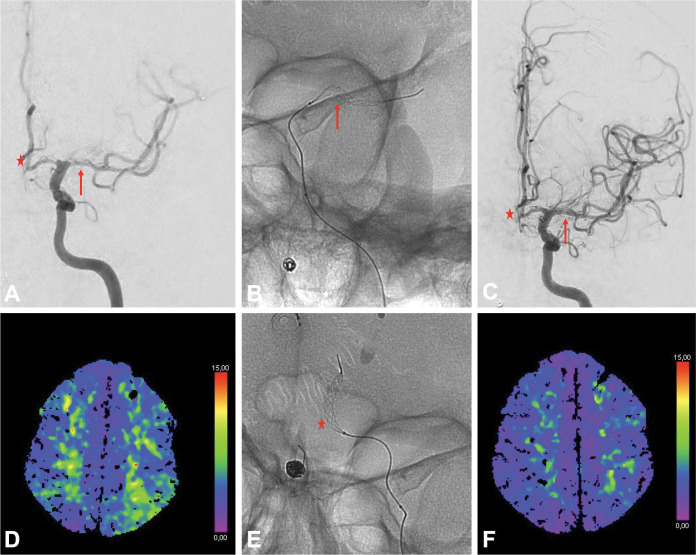
Cerebral angioplasty of the left middle cerebral artery (MCA) and left anterior cerebral artery (ACA) using the Comaneci device in a patient with a right V4/posteroinferior cerebellar artery aneurysm treated by coiling remodeling previously. **A.** Cerebral angiogram prior to endovascular treatment of the vasospasm demonstrating moderate vasospasm of the left A1 and A2 segments (star), as well as moderate vasospasm of the left M1 segment (arrow). **B.** Fluoroscopic image demonstrating angioplasty of the left M1 segment with the Comaneci (arrow). **C.** Final cerebral angiogram following angioplasty demonstrating an improved caliber of the left A1 (star) and left M1 (arrow) segments. **D.** Axial *T*_Max_ map before treatment showing a delayed *T*_Max_ for the left MCA territory. **E.** Fluoroscopic image demonstrating angioplasty of the left A2 segment with the Comaneci (star). **F.** Axial *T*_Max_ map following treatment showing an improved *T*_Max_ for the left MCA territory.

When inflated on a blank roadmap, the visibility of the Comaneci was described as excellent by the neurointerventionalists who performed the procedures in all cases (see Figures 1 and 2 for examples of the Comaneci).

## Discussion

In this study, we present our preliminary experience with the Comaneci device for the treatment of CV following aSAH. The angioplasty of 37 arterial segments (supraclinoid internal carotid artery; A1, A2, and A3 segments of the anterior cerebral artery; and M1 and M2 segments of the middle cerebral artery) was attempted, with a high success rate of 35/37 (95%). Post‑procedural outcomes indicated significant improvements in vessel diameter (+64%) and brain perfusion metrics (mean *T*_Max_ decreased by 45%). Importantly, there was no long‑term clinical complication, with only 6% per‑procedural complications, which were effectively managed.

These findings align with the latest AHA/ASA guidelines (2023), which suggest that endovascular interventions, such as cerebral angioplasty and intra‑arterial vasodilator therapy, can be considered reasonable options for patients with symptomatic CV unresponsive to hypertensive therapy (class IIa, level B) [18]. The guidelines emphasize the necessity for endovascular treatment to prevent acute ischemic stroke in cases where medical management fails. Despite this, there remains significant heterogeneity in the endovascular management of CV.

A recent large international survey of neurointerventionalists indicates that 58% of neurointerventionalists opt for endovascular treatment before maximizing medical management [9]. However, practical guidelines do not favor any specific endovascular technique over another, leading to diverse practices. Chemical angioplasty (e.g., calcium channel blockers) and mechanical angioplasty (using balloons or stent retrievers) are common, but only 17% of surveyed physicians view chemical intra‑arterial treatment as highly effective, while 65% prefer mechanical angioplasty [9]. Interestingly, 32% of neurointerventionalists believed that stentretriever angioplasty is more effective than chemical intra‑arterial treatment, while 11% disagreed and 57% were uncertain. Additionally, 27% considered balloon angioplasty more effective than stentretriever angioplasty, 13% disagreed, and 60% were unsure. Despite these perceptions, 79% of neurointerventionalists opted for chemical intra‑arterial treatment as the first line of action when medical management failed. This preference for chemical treatments over mechanical methods may stem from concerns about the risks associated with mechanical angioplasty [19], despite evidence supporting its safety and efficacy [21–24]. Moreover, there is a lack of familiarity with mesh angioplasty among the neurointerventional community, with more than half of physicians unable to provide informed feedback on these treatments. Additionally, physicians’ comfort levels with using mesh angioplasty vary, with some opting to treat only severe vasospasm and others willing to treat moderate cases.

The optimal timing and approach for aggressively treating symptomatic CV to prevent vasospasm‑mediated DCI remain subjects of ongoing debate. In this study, we describe our initial experience using the Comaneci device for the treatment of CV following aSAH. The Comaneci device demonstrated effectiveness in seven consecutive patients, treating 35 stenosed arterial segments with notable improvements in both angiographic and perfusion outcomes. When compared with specific semicompliant balloons, such as the Neurospeed balloon, the Comaneci showed slightly less improvement in vessel diameter (64% versus 81%) and reduction in brain hypoperfusion (45% versus 81% of the mean *T*_Max_) [25].

There were two procedural complications in our series, and those patients were not anticoagulated during the procedure. Those results encourage us to recommend systematically anticoagulating patients treated by mechanical angioplasty, as the recent survey among the neurointerventional community showed that 81% of physicians used IV per‑procedural heparin for those procedures [9]. The four principle assets of this device in the treatment of CV are its indication for use in intracranial stenosis contrary to stent retrievers used for acute ischemic stroke; its very good trackability, especially for the Comaneci 17, as it can fit 0.017” and 0.0165” microcatheters compared with balloons; the opened mesh, which permits the neurointerventionalist to inject IA supraselective medications while the Comaneci is opened contrary to balloons; and lastly, as we need a microcatheter to deliver the Comaneci, the same microcatheter could be used for rescue‑stenting in case of intracranial dissection during angioplasty.

The design of the Comaneci lends itself well to treat CV, and the risk of arterial perforation seems very low, as we did not experience it during our procedures. Previous studies have described a 1–4% risk of arterial rupture or dissection during angioplasty for the treatment of cerebral vasospasm; although, these studies were performed with earlier generations of angioplasty balloons.

At our institution, IA nimodipine is infused through the guiding catheter before and during cerebral angioplasty, which is supposed to maximize pharmacological vasodilation.

Despite the fact that patients with aSAH can develop DCI and lesions without visual angiographic vasospasm of large vessels [26, 27], CV remains an important cause of neurological complications after the aneurysmal treatment and may be responsible of approximately 20% of the global aSAH morbidity and mortality [28]. Approximately 30% of patients with CV will have neurologic deficits [29, 30].

Our study is not without limitations, and our findings are the results of an ongoing prospective observational study with inherent biases such as single‑center design and small sample size. Therefore, these findings are preliminary and need confirmation in larger studies. Manual corrections of the CTP core and hypoperfusion volumes were made by the same two senior neuroradiologists blinded to procedural outcomes. We did not use a core‑lab evaluation.

## Conclusions

This preliminary study demonstrates that compliant manually adjustable mesh, such as the Comaneci device, are effective in treating cerebral vasospasm following aSAH, offering good trackability and stability. Its low‑profile design may represent a technical advancement, potentially improving the safety and reliability of angioplasty, particularly in distal arteries, such as the A3 segments of the anterior cerebral artery.

## Figures and Tables

**Table:** 

CT	computed tomography
CTP	computed tomography perfusion
CV	cerebral vasospasm
DCI	delayed cerebral ischemia
IA	intra‑arterial
ICA	internal carotid artery
ICU	intensive care unit
mRS	modified Rankin Scale
NIHSS	National Institute of Health Stroke Scale
PTA	percutaneous transluminal angioplasty
aSAH	aneurysmal subarachnoid hemorrhage
T_max_	time to maximum
WFNS	World Federation of Neurological Society

## Additional File

The additional file for this article can be found as follows:

Supplementary File. Patients flow-chart.
