# Supplementary figures and images for: Treatment of Cerebral Vasospasm after Aneurysmal Subarachnoid Hemorrhage Using the Compliant Manually Adjustable Mesh Comaneci
Source: J Belg Soc Radiol. 2024 Oct 17;108(1):89. doi: 10.5334/jbsr.3714 (PMC11488188; doi:10.5334/jbsr.3714)

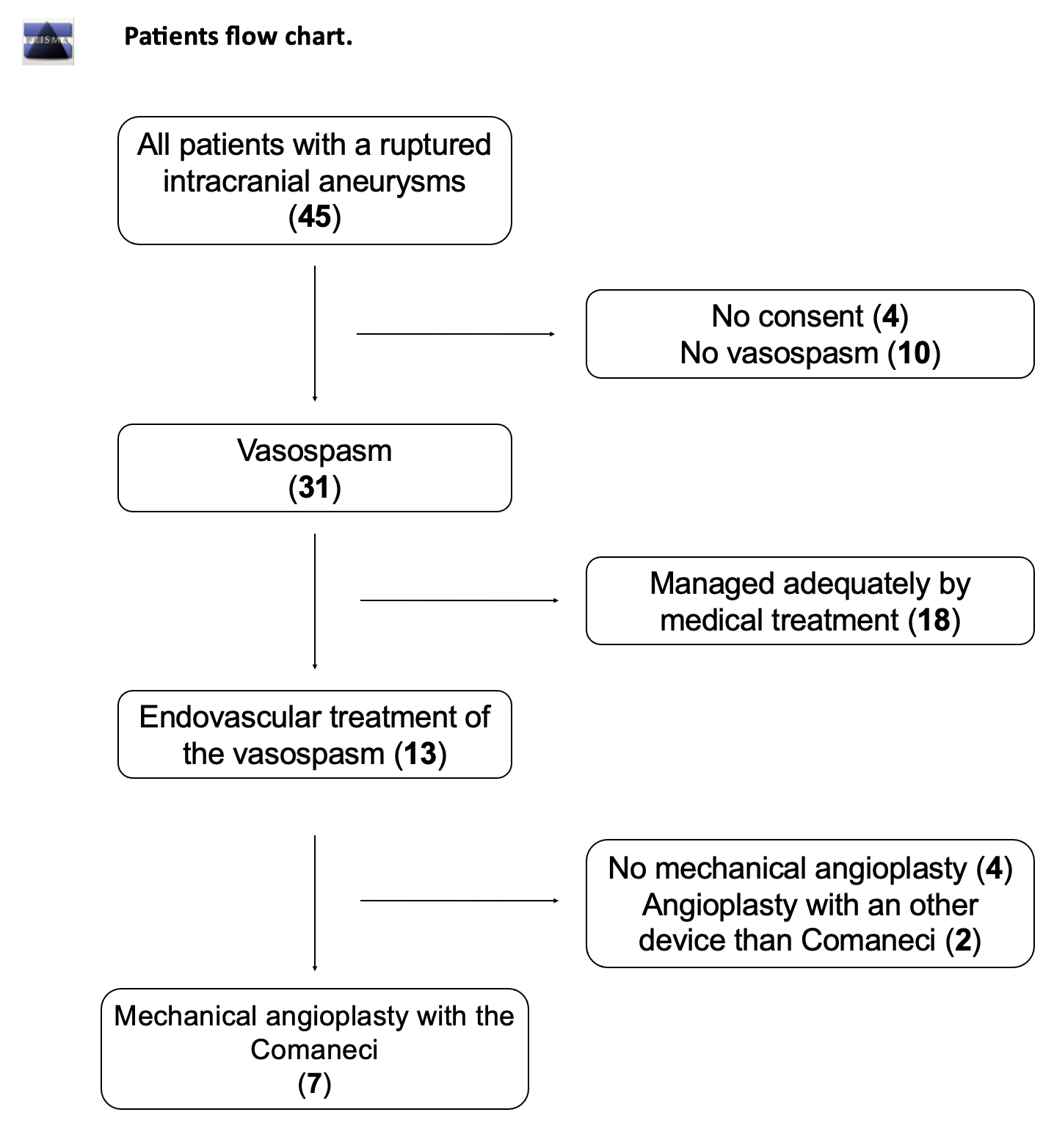

Supplement: Supplementary File. — Patients flow-chart. [file jbsr-108-1-3714-s1.tiff]
